# *In Situ*: Microbial Aerodynamic Microneedles for Targeted Drug Delivery Systems

**DOI:** 10.34133/research.0775

**Published:** 2025-08-12

**Authors:** Chenyu Zong, Fei Wang, Wenguo Cui

**Affiliations:** ^1^ Department of Orthopaedics, Shanghai Key Laboratory for Prevention and Treatment of Bone and Joint Diseases, Shanghai Institute of Traumatology and Orthopaedics, Ruijin Hospital, Shanghai Jiao Tong University School of Medicine, Shanghai 200025, PR China.; ^2^ Pharmaceutical Sciences Laboratory, Faculty of Science and Engineering, Åbo Akademi University, Turku 20520, Finland.

## Abstract

*In situ* microbial aerodynamic microneedles (MM-MNs) represent an autonomous transdermal drug delivery platform that utilizes the gas generated by microbial metabolism (e.g., H_2_, NO, and H_2_S) to propel drugs into deep tissues, surpassing the penetration limits of traditional microneedles reliant on external stimuli (heat/light/mechanical force). By leveraging controlled microbial metabolism, MM-MNs enable energy-independent, spatiotemporally precise delivery with enhanced targeting and bioavailability. Gas-driven propulsion combines with bioactive gas functions (e.g., NO-induced vasodilation and H_2_S-mediated anti-inflammation) to modulate disease microenvironments. The system’s biocompatibility (probiotic strains and *Lactobacillus*) and scalability (cost-effective patch design) further support its potential for localized therapies (skin diseases and tumors) with minimized systemic exposure. This innovation bridges microbial biotechnology and precision medicine, offering a paradigm shift in transdermal delivery.

Microbial aerodynamic microneedle (MM-MN) systems represent a breakthrough drug delivery technology that markedly improves the depth and targeting of drug delivery by combining gases generated by microbial metabolism with microneedle technology. Although traditional methods such as hyperthermia can enhance tissue penetration through physical diffusion, MM-MN systems exhibit unique advantages in multiple aspects. Firstly, MM-MNs do not rely on external energy devices such as lasers or ultrasound but instead rely on microbial metabolism to generate gases (such as H_2_ and NO) to drive drug delivery, greatly improving portability and long-term therapeutic applicability (such as chronic wound care). Secondly, compared with the risk that hyperthermia is vulnerable to the influence of tissue heterogeneity, which leads to uneven heat distribution, MM-MNs strictly limit the gas generation to the area of microneedle action through hydrogel encapsulation and strain metabolism regulation and use a short-half-life gas (such as NO, <5 min) to temporarily enhance the permeability of target blood vessels, achieving precise and controllable deep penetration, avoiding the diffuse damage of hyperthermia. Furthermore, MM-MNs not only enhance drug delivery efficiency through gas dynamics but also directly regulate the disease microenvironment by combining bioactive gases (such as NO promoting vasodilation and H_2_S being anti-inflammatory). For example, in the psoriasis model, the hydrogen-driven system simultaneously enhances calcipotriol permeation and inhibits local inflammation, while hyperthermia relies solely on a single physical mechanism. In addition, the system uses probiotics (such as lactic acid bacteria) and artificial intelligence (AI) to dynamically regulate the metabolic rate, combined with a thermosensitive microneedle barrier to prevent drug leakage, avoiding the risk of tissue overheating or degeneration that may be caused by hyperthermia. Finally, MM-MNs can be mass-produced as disposable patches with important cost-effectiveness, especially suitable for resource-limited scenarios. In summary, MM-MNs provide innovative solutions for deep drug delivery by integrating autonomous energy supply, synergistic biological functions, and precise control. MM-MN technology breaks through the limitation of traditional microneedle technology in the depth of drug delivery by using the gas generated by microbial metabolism to push drugs through the skin and into deep tissues. Traditional microneedle technology relies on external physical stimuli (such as light, heat, and mechanical force) to push drugs through the skin [1]. While MM-MNs provide a driving force through the natural metabolic processes of microorganisms, such as the production of hydrogen by *Escherichia coli* [2]. This innovative technology can substantially improve the depth of drug delivery. The advantage of an MM-MN system is that it does not need external energy support and relies entirely on the metabolic activities of microorganisms to drive drug delivery [3]. Thus, it provides a low-side-effect, efficient, and accurate treatment plan for precision medicine.

To visually illustrate this concept, Figure 1 presents a schematic overview of a microbial gas-powered microneedle system. As shown in the figure, gases produced by microbial metabolism provide the driving force to actively propel drugs through the skin, resulting in deeper and more efficient transdermal delivery compared to that of conventional microneedles. This innovative mechanism highlights the unique advantages of MM-MNs, including greater penetration depth, precise targeting, and energy independence.

**Figure 1.**
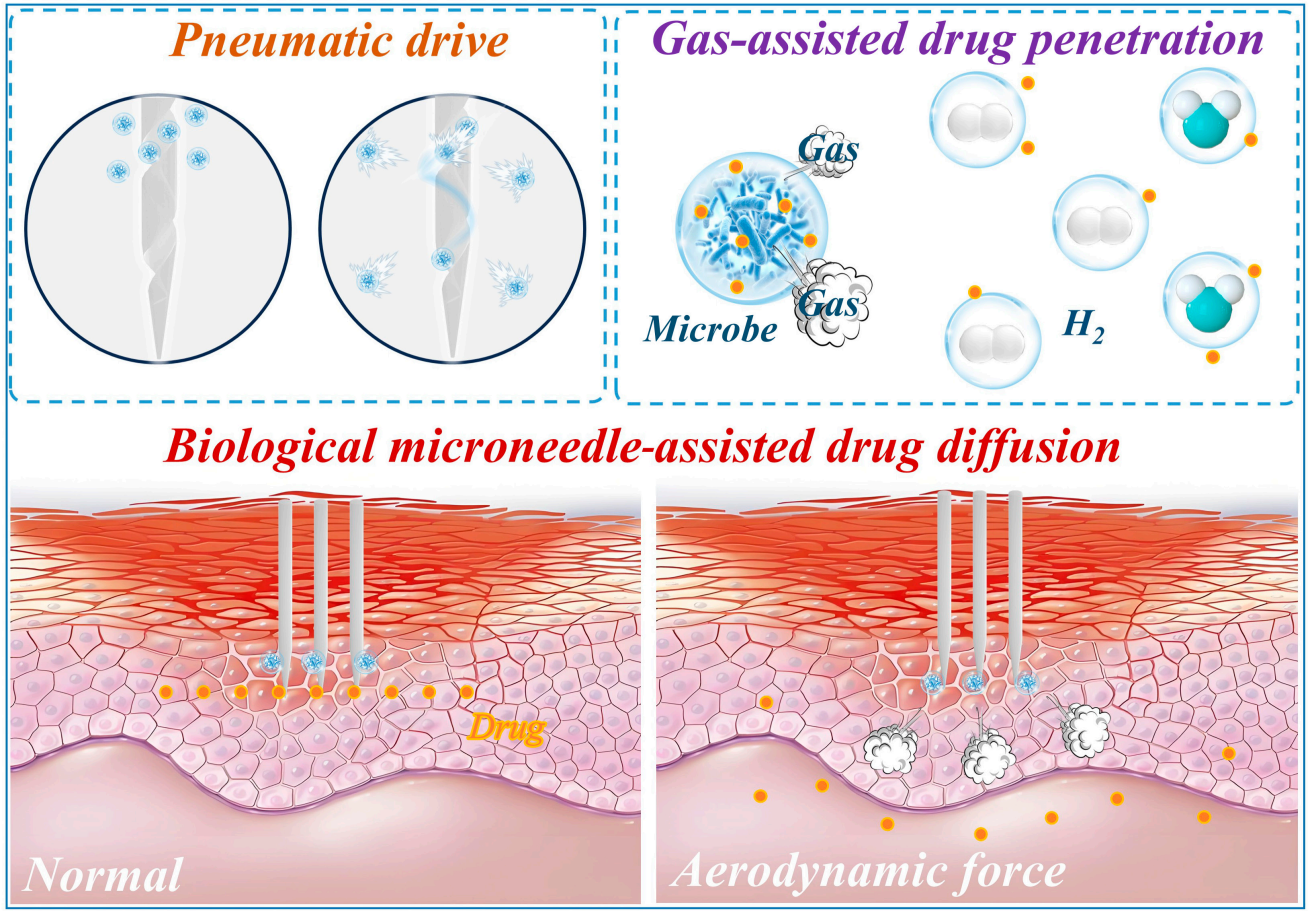
Acupuncture microneedles loaded with microbial aerodynamic hydrogel for drug delivery (artificial intelligence [AI] generation).

The working principle of MM-MN systems relies on the gas generated in the process of microbial metabolism [2]. For example, microorganisms such as *E. coli* produce hydrogen through their metabolic processes, and the pressure generated by this gas in the microneedle system can push drugs through the microneedles into the deep skin. Traditional microneedle delivery methods usually rely on the free diffusion of drugs through the epidermis and upper dermis. However, MM-MNs, driven by gas, can markedly improve the depth of drug delivery and achieve the penetration effect that traditional microneedle systems cannot achieve [4]. The advantage of this method is that through the metabolic gas production of microorganisms, the control of the drug delivery process no longer depends on external stimuli but is driven by the metabolic activities of microorganisms themselves, providing greater flexibility and control.

Another innovation of the MM-MN system is that it promotes the delivery of drugs through the generation of gas by microorganisms and uses the physical effect of gas in the delivery process to increase the concentration of drugs in the target area. Experimental data show that the depth of drug delivery can reach 1,000 μm using a gas-driven microneedle system, which exceeds the delivery depth of the traditional microneedle system [3]. This development not only significantly improves the concentration of drugs in the target area but also improves the accuracy of treatment, especially in the field of treatment requiring precise drug delivery, such as skin diseases and local cancer [3].

Microorganism-driven gas production capacity is one of the core strengths of the system. Studies have shown that *Enterobacter aerogenes* (*E. coli*) can be effectively encapsulated in microneedles and continuously produce gas within hours. The generation of this gas drives the dissolution and release of the drug, and the sustained gas driving force can reach more than 0.04 N, which is enough to push the drug through the deep skin. Compared with traditional metal-based microneedle materials, the microorganism-driven gas system can continuously provide a strong driving force and maintain a stable drug release rate and gas generation capacity [3]. The continuous gas dynamics of the system can provide long-term impetus for drugs, ensure that drugs can be precisely delivered to deep tissues, and overcome the depth limitations that traditional microneedles cannot achieve. In addition, the gases produced during microbial metabolism are not limited to hydrogen but may also include other gases that can further enhance the effectiveness of drug delivery.

Compared to chemical-gas-producing microneedles, MM-MNs achieve sustainable and self-powered drug driving through microbial metabolism. For example, although an acid–base reaction system (NaHCO_3_/H^+^ → CO_2_) can produce gas briefly, it cannot be sustained after the reagents are exhausted [5], while engineered lactic acid bacteria can continuously generate NO using nitrate in the host microenvironment (>24 h) [6]. In addition, microbial systems achieve dynamic controllability through synthetic biology tools such as inducible promoters and quorum sensing, while chemical reaction rates are susceptible to environmental fluctuations [5]. In terms of biocompatibility, MM-MNs avoid the irritating by-products of chemical systems (such as strong acids) and directly intervene in disease progression using the therapeutic function of gas itself (such as H_2_S anti-inflammatory) [6], while CO_2_ serves only as a physical driving force and may even exacerbate local acidification [7]. These differentiated advantages highlight the unique value of MM-MNs in precision medicine.

In terms of gas selection, besides hydrogen, MM-MN systems can also learn from the characteristics of other bacteria and gases to further enhance the effect of drug delivery. Certain bacteria are capable of producing nitric oxide (NO) and hydrogen sulfide (H_2_S), gases that play important physiological roles in the body. Nitric oxide helps to improve the bioavailability of drugs by dilating blood vessels, promoting blood circulation, and regulating immune response. In addition, nitric oxide can also promote the increase of local blood flow, thus improving the delivery efficiency of drugs. Hydrogen sulfide plays an important role in anti-inflammation, neuro-protection, and tissue repair, which can enhance the targeting of drugs in the target area and improve the therapeutic effect. By selecting suitable bacterial species and optimizing their metabolic processes, the MM-MN system is able to utilize these gases more efficiently, further improving the efficacy of drug delivery.

The MM-MN system leverages diverse microbial gases to achieve multifunctional therapeutic effects. As summarized in Table 1, hydrogen (H_2_) generated by *E. coli* provides both propulsion force and antioxidant support, while nitric oxide (NO) from engineered *Lactobacillus* enhances vascular permeability and immune modulation. Hydrogen sulfide (H_2_S), produced by modified *Saccharomyces cerevisiae*, further augments the anti-inflammatory and regenerative effects. These gases synergize with drug payloads to address complex disease microenvironments (e.g., psoriasis and tumors), surpassing the single-mechanism limitations of traditional approaches.

**Table 1.** Comparison between MM-MNs and chemical gas production microneedles

Compared dimensions	MM-MNs	Chemical gas production microneedles
Sustainability	Microorganisms continuously produce gas by metabolizing substrates such as glucose and nitrate, and the gas generation is synchronized with bacterial activity (up to 48 h) [3]	Gas production relies on limited chemical reagents (such as NaHCO_3_ + H^+^), and gas depletion occurs after a single reaction [5]
Controllability	AI real-time regulation [16] or an inducible promoter (such as pH/hypoxia response) dynamically regulates gas production rate and duration [6,11]	The gas production rate is limited by the concentration of reactants and environmental conditions (such as pH and temperature), making it difficult to accurately regulate [5]
Biocompatibility	Using probiotics (such as lactobacilli) and biocompatible gases (NO and H_2_S), without irritating by-products [6,8]	Strong acid/strong alkali reactions may cause local tissue irritation (such as residual HCl), and excessive accumulation of CO_2_ may trigger inflammation [5,7]
Functional integration	Gas combines power drive and biological activity (such as NO promoting vasodilation and H_2_S being anti-inflammatory), synergistically enhancing therapeutic efficacy	Gas (such as CO_2_) serves only as a physical driving force and lacks therapeutic function [5]
Conversion potential	Can be designed as a disposable patch, without the need for external reagent supplementation. Bacterial self-destruct switch [11] ensures clinical safety	Preloaded chemical reagents are required, and long-term storage stability is limited; Residual chemicals may require additional removal steps [7]

MM-MNs, microbial aerodynamic microneedles

For example, safe microorganisms such as *Lactobacillus* and *S. cerevisiae* can be genetically modified to continuously produce nitric oxide or hydrogen sulfide, which can help enhance the targeting and bioavailability of drugs. Some strains of *Lactobacillus* can produce nitric oxide by reducing nitrate or nitrite, which not only h elps to dilate blood vessels but also increases the concentration of drugs in local tissues, especially in the treatment of diseases that need to promote local blood flow or immune regulation, which can further improve the efficacy of drugs. At the same time, *S. cerevisiae* and other strains can produce hydrogen sulfide by reducing sulfate, which plays an important role in anti-inflammatory, anti-tumor, and neuro-protection effects. The microbial gases, their sources, physiological roles, and proposed therapeutic functions in MM-MN systems are summarized in Table 2.

**Table 2.** Microbial gases in MM-MNs: sources, physiological roles, and therapeutic functions

Microbial gas	Microbial source	Physiological role	Proposed function in MM-MN systems	References
Hydrogen (H_2_)	*Escherichia coli* (natural producer)	Antioxidant, anti-inflammatory	Drives drug propulsion via gas pressure, enhances local tissue repair, and reduces oxidative stress	[3,6]
Nitric oxide (NO)	Engineered *Lactobacillus* (via nitrate reduction)	Vasodilation, immune modulation, antimicrobial activity	Enhances drug bioavailability by improving local blood flow; synergizes with anti-inflammatory drugs	[8,16]
Hydrogen sulfide (H_2_S)	Engineered *Saccharomyces cerevisiae* (via sulfate reduction)	Anti-inflammatory, neuro-protective, promotes angiogenesis	Amplifies drug targeting in inflamed/tumor tissues; supports tissue regeneration	[6,11]

In terms of clinical applications, MM-MN systems have shown a wide range of potential, especially in the field of chronic disease treatment and local cancer treatment. MM-MNs can provide deeper and more efficient drug delivery, breaking through traditional methods’ limitations. For example, in the treatment of skin diseases such as psoriasis, microbe-driven drug delivery can not only improve drug permeability [8]. It can also reduce the side effects of drugs in the body, thereby improving the treatment compliance and quality of life of patients. In an experiment, calcipotriol was successfully delivered to a deeper layer of the skin via a hydrogen-driven microneedle system, notably relieving the symptoms of psoriasis and effectively reducing side effects [6]. In addition, MM-MN systems can precisely deliver anticancer drugs to tumor tissues to reduce toxic and side effects to normal tissues, showing great potential in local cancer therapy.

To mitigate biosafety concerns, the MM-MN system now employs engineered *Lactobacillus* and *S. cerevisiae* as chassis. These strains are equipped with inducible promoters for context-dependent gas synthesis (e.g., pH-responsive NO production) and programmed kill switches to ensure self-elimination posttreatment. Such strategies align with Food and Drug Administration guidelines for live biotherapeutic products, enhancing clinical translatability while retaining gas-driven therapeutic efficacy.

However, although the MM-MN system exhibits significant advantages, it still faces some challenges. First, the selection and optimization of microorganisms remains a key issue [9]. Although *E. coli* can produce hydrogen efficiently, its potential pathogenicity and instability in different environments may pose a risk to clinical applications [10]. Therefore, future research needs to focus on the selection of safer and more stable microbial strains, such as genetically modified lactobacilli or yeasts, for better security and stability. Second, the precise release control of gas is still one of the difficulties in technical optimization [5]. How to precisely control the release rate and amount of gas in order to better control the delivery of drugs is still the key to improving system stability and therapeutic effect. Future research can optimize the gas permeability of microneedle materials or combine smart response materials to regulate the metabolic process of microorganisms [11]. Precise control of gas release is achieved.

Except for the precise delivery of MM-MNs, intradermal gas control behavior is also crucial. To solve the problem of intradermal gas control, the MM-MN system ensures accurate regulation through a multilayer strategy. Firstly, the microneedles use gas-permeable hydrogel materials with adjustable porosity and limit the gas diffusion range by adjusting the cross-linking density to achieve localization. Secondly, the metabolic rate of genetically modified probiotic strains (such as lactic acid bacteria and brewing yeast) is matched with drug release kinetics, and the gas generation area is strictly restricted by microenvironmental conditions (pH and nutrient supply). Additionally, the AI-driven real-time monitoring system combines sensors and machine learning algorithms to dynamically regulate metabolic activity, maintaining gas pressure within a preset threshold and ensuring that the depth of gas action is limited to the dermis layer. Regarding the safety risks of nonsystemic drugs, the system mitigates them through the following measures: calibrating the microneedle length to 800 to 1,000 μm and verifying through *in vitro* pig skin experiments that 94% of drugs remain in the dermis layer. The microneedle tip introduces a thermosensitive polymer barrier that dissolves only upon contact with the skin to prevent drug penetration into the systemic circulation. At the same time, microbial gases (such as NO and H_2_S) have a short half-life (<5 min) and are rapidly metabolized, further anchoring therapeutic effects with local vasodilation and anti-inflammatory effects. A preclinical mouse model showed that the systemic drug concentration was below the detection limit, confirming local safety. In the future, the safety of bacterial strains (such as replacing them with bifidobacteria) will be optimized and pH-responsive materials will be developed to dynamically block the generation of gases that penetrate unexpectedly deep.

In the process of drug delivery, in addition to the dynamic action of driving drugs, the consumption of gases by microorganisms can also provide unique therapeutic effects [12]. For example, through the consumption of gas by bacteria, not only can drug delivery be promoted, but also negative-pressure suction can be achieved to further enhance the therapeutic effect. In the traditional vacuum aspiration technique, negative pressure is created by external equipment to promote local blood circulation, remove wound secretion, and accelerate cell regeneration. An MM-MN system can break the dependence of traditional negative-pressure suction technology on equipment and site and provide more efficient, flexible, and convenient treatment methods [7]. Gas consumption during bacterial metabolism can generate negative pressure, further promote local tissue repair, and enhance the depth and durability of drug delivery. In this way, drug delivery and local tissue repair can be performed simultaneously, improving the therapeutic effect [13]. It is especially suitable for wound healing and chronic disease treatment [14].

The involvement of AI in the MM-MN system is also significant [15]. AI technology can accurately regulate the drug delivery process by monitoring the dynamic changes of microbial metabolism in real time, combining with gas release rate, drug delivery depth, and other variables [16]. AI can track the generation and release of gases in real time with the help of sensors and automatically adjust the metabolic activities of microorganisms through machine learning algorithms to ensure the accuracy and consistency of drug delivery. Through real-time feedback and intelligent control, AI technology will be able to optimize microbial metabolic processes, achieve precise control of drug delivery, and provide more personalized and efficient treatment options.

The acupuncture-microneedle-loaded hydrogel microsphere system proposed by Professor Wenguo Cui provides design implications for MM-MN systems, especially in terms of drug delivery depth and breadth [17–19]. The acupuncture microneedle system promotes the depth and breadth of drug delivery through the pneumatic force generated by microorganisms, which can achieve precise drug delivery in a wider range and provide a new idea for the clinical application of MM-MN systems. Combining the generation of microbial metabolic gases and negative pressure suction effects, MM-MN systems may be able to simultaneously achieve drug delivery and local tissue repairin the future, further enhancing therapeutic efficacy [20]. By doing so, it will further promote the widespread application of MM-MN.

To sum up, MM-MN systems rely on their innovative drug delivery mechanism, precise drug controlled release capability, and microbial metabolism-driven mechanism [21]. They show great potential for clinical application. The system not only breaks through the limitations of traditional microneedles but also provides a more flexible and accurate drug delivery scheme through the gas metabolism of microorganisms. Despite the challenges of microbial selection, safety control, and gas release regulation, MM-MNs are expected to become an important tool in the fields of precision medicine and multimodal therapy, with the continuous development of microbiology, materials science, and drug delivery technology, which are widely used in chronic diseases, cancer treatment, and immunotherapy [22]. Combined with negative-pressure suction and intelligent response technology, MM-MN systems may provide more efficient, safe, and personalized treatment for patients [23]. To further promote the development of precision medicine and bring better treatment effect and experience to patients globally, further research and clinical implementation are essential.

## References

[1] He G, Li YS, Younis MR, Fu LH, He T, Lei S, Lin J, Huang P. Synthetic biology-instructed transdermal microneedle patch for traceable photodynamic therapy. Nat Commun. 2022;13(1): Article 6238. 36266306 10.1038/s41467-022-33837-1 PMC9585024

[2] Wang X, Cheng W, Su J. Research progress of extracellular vesicles-loaded microneedle technology. Pharmaceutics. 2024;16(3): Article 326. 38543220 10.3390/pharmaceutics16030326 PMC10975918

[3] Zheng B, Li QY, Fang LP, Cai XL, Liu Y, Duo YH, Li BW, Wu ZY, Shen BX, Bai Y, et al. Microorganism microneedle micro-engine depth drug delivery. Nat Commun. 2024;15(1): Article 8947. 39414855 10.1038/s41467-024-53280-8 PMC11484856

[4] Fang A, Wang YF, Guan NY, Zuo YM, Lin LM, Guo BJ, Mo AS, Wu YL, Lin XR, Cai WX, et al. Porous microneedle patch with sustained delivery of extracellular vesicles mitigates severe spinal cord injury. Nat Commun. 2023;14(1): Article 4011. 37419902 10.1038/s41467-023-39745-2 PMC10328956

[5] Yu L, Hu P, Chen Y. Gas-generating nanoplatforms: Material chemistry, multifunctionality, and gas therapy. Adv Mater. 2018;30(49): Article e1801964. 30066474 10.1002/adma.201801964

[6] Kim H, Lee J, Heo U, Jayashankar DK, Agno KC, Kim Y, Kim CY, Oh Y, Byun SH, Choi B, et al. Skin preparation-free, stretchable microneedle adhesive patches for reliable electrophysiological sensing and exoskeleton robot control. Sci Adv. 2024;10(3): Article eadk5260. 38232166 10.1126/sciadv.adk5260 PMC10793950

[7] Ye Y, Yu J, Wen D, Kahkoska AR, Gu Z. Polymeric microneedles for transdermal protein delivery. Adv Drug Deliv Rev. 2018;127:106–118. 29408182 10.1016/j.addr.2018.01.015 PMC6020694

[8] Chen W, Tian R, Xu C, Yung BC, Wang GH, Liu YJ, Ni QQ, Zhang FW, Zhou ZJ, Wang JJ, et al. Microneedle-array patches loaded with dual mineralized protein/peptide particles for type 2 diabetes therapy. Nat Commun. 2017;8(1): Article 1777. 29176623 10.1038/s41467-017-01764-1 PMC5701150

[9] Lin K, Gueble SE, Sundaram RK, Huseman ED, Bindra RS, Herzon SB. Mechanism-based design of agents that selectively target drug-resistant glioma. Science. 2022;377(6605):502–511. 35901163 10.1126/science.abn7570 PMC9502022

[10] Meng J, Jin ZK, Zhao PH, Zhao B, Fan MJ, He QJ. A multistage assembly/disassembly strategy for tumor-targeted CO delivery. Sci Adv. 2020;6(20): Article eaba1362. 32440551 10.1126/sciadv.aba1362 PMC7228751

[11] Zhao PH, Jin ZK, Chen Q, Yang T, Chen DY, Meng J, Lu XF, Gu Z, He QJ. Local generation of hydrogen for enhanced photothermal therapy. Nat Commun. 2018;9(1): Article 4241. 30315173 10.1038/s41467-018-06630-2 PMC6185976

[12] Chen S, Yu Y, Xie S, Liang D, Shi W, Chen S, Li G, Tang W, Liu C, He J. Local H_2_ release remodels senescence microenvironment for improved repair of injured bone. Nat Commun. 2023;14(1): Article 7783. 38012166 10.1038/s41467-023-43618-z PMC10682449

[13] Zeng J, Zhao XA, Liang Z, Hidalgo I, Gebert M, Fan PF, Wenzl C, Gornik SG, Lohmann JU. Nitric oxide controls shoot meristem activity via regulation of DNA methylation. Nat Commun. 2023;14(1): Article 8001. 38049411 10.1038/s41467-023-43705-1 PMC10696095

[14] Joulia E, Metallo CM. Methionine and H_2_S alter cancer-immune dialogue. Nat Metab. 2023;5(9):1456–1458. 37537368 10.1038/s42255-023-00862-3

[15] Yang Z, Gao D, Zhao J, Yang GJ, Guo M, Wang Y, Ren XC, Kim JS, Jin L, Tian ZM, et al. Thermal immuno-nanomedicine in cancer. Nat Rev Clin Oncol. 2023;20(2):116–134. 36604531 10.1038/s41571-022-00717-y

[16] He ZS, Chen W, Hu K, Luo YY, Zeng WQ, He X, Li TT, Li YJ. Resolvin D1 delivery to lesional macrophages using antioxidative black phosphorus nanosheets for atherosclerosis treatment. Nat Nanotechnol. 2024;19:1386–1398. 38898135 10.1038/s41565-024-01687-1 PMC12871472

[17] Lin F, Xiang L, Wu LX, Liu YP, Jiang QZ, Deng LF, Cui WG. Positioning regulation of organelle network via Chinese microneedle. Sci Adv. 2024;10(16): Article eadl3063. 38640234 10.1126/sciadv.adl3063 PMC11029808

[18] Lin F, Zhuang YP, Xiang L, Ye TJ, Wang Z, Wu LX, Liu YP, Deng LF, Cui WG. Localization of lesion cells and targeted mitochondria via embedded hydrogel microsphere using heat transfer microneedles. Adv Funct Mater. 2023;33(18): Article 2212730.

[19] Lin F, Wang Z, Xiang L, Wu LX, Liu YP, Xi XB, Deng LF, Cui WG. Transporting hydrogel via Chinese acupuncture needles for lesion positioning therapy. Sci Adv. 2022;9(17): Article e2200079. 10.1002/advs.202200079 PMC9189641 35404511

[20] Yang L, Zhang D, Li WJ, Lin HB, Ding CD, Liu QY, Wang LL, Li ZM, Mei L, Chen HZ, et al. Biofilm microenvironment triggered self-enhancing photodynamic immunomodulatory microneedle for diabetic wound therapy. Nat Commun. 2023;14: Article 7658. 37996471 10.1038/s41467-023-43067-8 PMC10667311

[21] Li X, Hu Y, Zhang XC, Shi XY, Parak WJ, Pich A. Transvascular transport of nanocarriers for tumor delivery. Nat Commun. 2024;15(1): Article 8172. 39289401 10.1038/s41467-024-52416-0 PMC11408679

[22] Zhang X. AI-recognized mitochondrial phenotype enables identification of drug targets. Nat Comput. 2024;4(8):563–564. 10.1038/s43588-024-00682-9 39174760

[23] Yu M, Li WM, Yu YR, Zhao Y, Xiao LZ, Lauschke VM, Cheng YY, Zhang Y, Wang Y. Deep learning large-scale drug discovery and repurposing. Nat Comput. 2024;4(8):600–614. 10.1038/s43588-024-00679-4 39169261

